# Will extended field-of-view PET/CT depopulate the graveyard of failed PET radiopharmaceuticals?

**DOI:** 10.1186/s40644-022-00510-1

**Published:** 2022-12-19

**Authors:** E. F. J. de Vries, P. H. Elsinga, C. Tsoumpas

**Affiliations:** Department of Nuclear Medicine and Molecular Imaging, University Medical Center Groningen, Hanzeplein 1, Groningen, 9713GZ The Netherlands

**Keywords:** PET-radiopharmaceutical, Extended field of view, PET-camera, Sensitivity

## Abstract

With the rapid emergence of extended Field-of-View PET-cameras several new applications for radiopharmaceuticals become within reach. Main reason is the significant increase of the sensitivity of the PET-camera so that much less radioactivity can be administered. Issues that that hampered development or use of PET-radiopharmaceuticals become realistic again. Molar activity requirements can become less strict. New low-yielding radiochemistry methods may become applicable. Carbon-11 labelled compounds can revive and potentially be shipped to nearby PET-facilities. PET-radiopharmaceuticals with slow kinetics in comparison to their half life can still be used. As additional infrastructure and equipment will likely remain unchanged and keep the same sensitivity therefore there will be issues with kinetic modelling requiring analysis of plasma or metabolites samples with lower count rate. Besides the potential revival of failed radiopharmaceuticals, novel challenges are ahead to develop novel radiochemistry based on thus far unsuitable (low yielding or time consuming) reactions.

## Introduction

With the rapid emergence of long axial field of view positron emission tomographs (PET) a wide range of new opportunities has become available in the field of nuclear medicine [1–4]. A collective combination of these opportunities can boost the development of new radiotracers. The increase of whole-body sensitivity can be as high as a factor of 10 for 1 m long PET and 40 for a 2 m long PET scanner [5–7], though it is important to mention that these numbers can vary depending on the patient and tracer distribution.

The extension of the axial field of view of the PET scanners provides a combination of the following advantages:

- Improved image quality and high signal to noise
- Imaging with lower injected doses
- Imaging kinetics with greater temporal range
- Imaging longer axial FOV (LAFOV)

In this paper it is described how each of these advantages may recreate interest in previously failed PET radiotracers.

## New features of extended field-of-view PET cameras

### Improved image quality and high signal to noise

As is shown recently with images from ^89^Zr monoclonal antibodies [8], the superior sensitivity of LAFOV PET can provide impressive images even for radionuclides with relatively low branching ratio of positron emission. Such radionuclides are ^64^Cu, ^76^Br, ^86^Y, and ^124^I. The latter can be very useful in translating successful SPECT imaging tracers radiolabelled with ^123^I into PET.

Another consequence of these ultrahigh sensitivity PET scanners is the potential exploration of novel ideas originating from fundamental positron physics. The fact that the positron lifetime depends on the surrounding materials is well known and practically used in other scientific fields. Scientists have been able to demonstrate a potentially practical method to measure the positron’s lifetime in vivo and thus characterise its surroundings (e.g., hypoxic levels) [9]. This can be achieved by any positron emitter which simultaneously to the positron emits an additional photon, which occurs in several nuclides (e.g., ^94^Tc, ^44^Sc, ^124^I). The additional photon is used to setup the ‘timer’ of the lifetime of the positron and this life-time is indicator of paramagnetic material concentrations, such us oxygen. The increased sensitivity of the scanners can help illustrate if such use of PET scanners is useful and practically meaningful.

### Enabling imaging more than one tracer simultaneously

The supreme improvement in the signal measured may allow accurate kinetic/temporal separation of the two images when two tracers are scanned simultaneously. This is an approach that in principle has been developed previously, but not translated in the clinical practice due to lack of consistent and stable separation of the signal from the two radiotracers. The use of two tracers simultaneously could allow to measure and correlate different biological processes at the same time and position, which in addition will simplify several logistical challenges in maximising the use of the relatively limited capacity and number of extended FOV PET scanners. Such examples are the potential simultaneous imaging of [^18^F]FDG with FAP or PSMA binding PET-tracers or with radionuclides used for radionuclide therapy in a way of measuring the efficacy of treatment at voxel level [10].

### Imaging with lower injected doses

The advantage of high sensitivity can be exchanged by reducing the amount of radioactivity of the injected radiotracer. Even poor labelling efficiency or low positron emission branching ratio would not be such an issue and more radionuclides and radiotracers can be considered. Another advantage that the low dose can bring is the wider acceptance for use in clinical trials. When imaging with an extended FOV PET-camera, an at least 8–10 times reduction of the injected dose is expected to produce equivalent or even better images than the current state-of-the-art PET scanners. This would correspond to making PET scanning available to healthy volunteers [11, 12] – as long as the CT image is only necessary for attenuation correction. Another consequence of dose reduction is the possibility of imaging the same patient during more sessions, which leads to the use of PET more regularly as a tool for accurate therapy evaluation and planning.

### Imaging kinetics with greater temporal range

Perhaps this is the most exciting opportunity in terms scientific investigations as the high sensitivity of the scanners enables imaging both slower and faster biological processes [13]. Images of 1 s duration can have a reasonable image quality and can help in providing information of faster biological processes [14, 15]. This is a widely unexplored area in PET imaging. Likewise, imaging biological processes for much longer half-lives of the radiotracer has not been possible until the development of total-body PET. One order increase of sensitivity can be translated up to 4–5 half-lives after injection with standard imaging protocols, but longer half-lives (e.g. 5–6) are also feasible if one extends the scanning duration protocol accordingly. For example, this would effectively mean that a monoclonal antibody labelled with ^89^Zr could be traced in the body three to four weeks after its administration [16], or a nanobody labelled with ^18^F could be traced even 11 h after injection.

### Imaging longer axial FOV

Perhaps at a lesser extent, imaging multiple regions at the body may provide incidental interesting findings related to the radiotracer behaviour in other areas of the body beyond the one of interest – something that currently some groups are calling the body axes. These may be related to brain-heart-liver-kidneys or brain-gut axes. Simultaneous dynamic imaging of multiple organs, or even the same large organs (spinal cord) as well as looking to a person as a whole (e.g., cardiovascular system, musculoskeletal system) rather than in parts may provide new insights and findings which could revolutionise medicine and bring it closer to a “systems biology” approach of the humans. This can help the molecular understanding of systemic and interrelated diseases and disorders.

What is the obvious use of a longer scanner is the in vivo biodistribution measurement of any newly developed radiotracers or even potentially radiolabelled drugs including those assessing the efficacy of radionuclide or other therapies.

## Consequences of new extended FOV features for application of radiopharmaceuticals

A lower dose of radioactivity as a consequence of increased sensitivity of the PET-camera can be allowed resulting in high quality PET-images and effecting:

### Molar activity requirements

To detect sufficient PET-signal in a voxel of about 4–10 mm^3^, a minimal required number of counts needs to be detected from the corresponding tissue. Simultaneously the molecular target of investigation should not be saturated by injected cold radioligand as this sets a limit to the binding of the radioligand. So, the ratio of the radioactivity and the mass of the radioligand in the voxel corresponds to a certain minimal molar activity (*A*_*m*_). As a result of the increased sensitivity of the extended FOV camera the minimal required radioactivity per voxel will become even smaller. Consequently, first the requirements for production of PET-radioligands with high *A*_*m*_ will become less demanding; second, the productions with longer synthesis times that result in lower *A*_*m*_ because of radioactive decay can still deliver radioligand with suitable quality; and finally the timing of delayed injections may become more flexible as like the previous argument radioactive decay lowering *A*_*m*_ is less of an issue.

Maintaining the high *A*_*m*_ for radioligands (as the situation before introduction of extended FOV cameras) will enable PET-studies of molecular targets with lower densities as with decreased amount of radioactivity injected the administered mass will decrease resulting lower target occupancy. As Am is a fixed ratio of radioactivity over mass, by injecting a lower dose both the number of counts (radioactivity) is decreased together with the injected mass enabling imaging of low-density targets without affecting its occupancy. An additional advantage is that a lower *A*_*m*_ may be allowed for radioligands with an extremely high toxicity as compared to the traditional situation and that injection of a lower dose (and thus lower mass) may relieve toxicity issues.

In practice the less strict requirement to obtain high *A*_*m*_ will mainly affect ^11^C-radioligands as their *A*_*m*_ is usually lower than for other radionuclides. To achieve high *A*_*m*_ for ^11^C-radioligands is very demanding as introduction of cold carrier 12C can easily happen and lower the A_m_. Naturally occurring carbon dioxide or methane is present in the atmosphere being the non-radioactive analogues of the standard ^11^C-starting compounds [^11^C]CO_2_ and [^11^C]CH_4_ produced by the cyclotron. These non-radioactive carriers need to be excluded from the synthesis system as these will be converted to the non-radioactive radioligand in the same batch [17].

### Production methods with low radiochemical yield become into reach again

Several low-yielding production methods, including multi-step radiolabelling procedures that required long reaction times as compared to the half-life of the radionuclide such as multi-component reactions [18] or gave low radiochemical yields [19] might revive as the minimal required injected radioactivity dose. Example of a low yielding production method was published by Antunes et al. who produced the promising androgen receptor tracer [^18^F]enzalutumide. Though preclinical results were very promising, activity yields were that low that human studies were not possible. Examples of low-yielding reactions are multicomponent reactions and Diels-Alder reactions. Currently such methods have been abandoned or are solely used by a few highly trained research groups. This case may be especially true for ^11^C. It may also become possible to translate cold organic chemistry reactions that need longer reaction times into PET-radiolabelling procedures. One could also compromise on the use of less (expensive) precursor for radiolabelling allowing lower radiochemical yields being still enough for extended FOV PET-cameras.

### ^11^C-Radiopharmaceuticals may gain popularity again and can be shipped over longer distances

Regarding the small molecule-based radiotracers, ^18^F-radiotracers are gradually taking over the ^11^C-analogs because the longer half-life of 110 min makes ^18^F-radiotracers more convenient from logistics point of view [20–23]. On one hand, patient planning in a Nuclear Medicine department with ^18^F-tracers is easier with respect to radiosynthesis production timing. Also, in most cases sufficient ^18^F-radiotracer can be produced to study multiple patients on the same day. In addition, timing of injection of the radiotracer is more flexible. On the other hand, ^18^F-radiotracers are also easier to distribute to other hospitals that do not have their own cyclotron as a result of the longer half-life as compared to ^11^C. This has also increased the commercial value of ^18^F-radiotracers. As a result, several companies have adopted ^18^F-radiotracers in their portfolio, and acquired a marketing authorisation. However, since the extended FOV-cameras require much less radioactivity, it can become easier to also distribute ^11^C-radiotracers to other hospitals. Assuming an increased sensitivity of 8-fold, an additional hour (3x the half-life) needed for transportation could be acceptable to acquire a similar radioactivity signal compared to normal FOV cameras.

### New possibilities for tracers that showed too slow kinetics compared to half-life of the radionuclide

In general, PET tracers should be labelled with a radionuclide with a half-life that matches the kinetics of the tracer. If the half-life of the radionuclide is too short, radioactive decay will not leave sufficient time for the unbound tracer to be cleared from circulation and tissues of interest, which will preclude the acquisition of images with a high contrast between specifically bound tracer at the target site and the nonspecific background signal. In some cases, tracers can be labelled with an isotope with a longer half-life, but in other cases this will not be useful. All organic compounds contain carbon atoms that – at least theoretically – can be substituted for a ^11^C isotope without changing its chemical and biological properties. Therefore ^11^C-labelled compounds can be used in drug development to assess the administration, distribution, metabolism and excretion to obtain a better understanding of its biodistribution and kinetics of a newly developed drug over a longer period of time by using the extended FOV PET-camera. Usually such information is obtained from blood or urine samples. Many drugs contain a fluorine atom that can be replaced with an ^18^F isotope. Labelling such compounds with a longer-lived isotope of an atom that is not naturally present in the molecule will change the molecular structure and could compromise the binding properties. Alternatively, the structure of the molecule could be adjusted to increase the clearance rate, but this will also affect the binding properties. In both cases, the relative impact of structural modifications is larger for small molecules than for large biomolecules like proteins. Even in cases where a long-lived isotope can be used to compensate for slow tracer kinetics, this comes with a cost: it will expose the patient to higher radiation burden, which may not be acceptable for all applications.

In cases where only a static PET scan is required, the enhanced sensitivity of extended field-of-view scanners allows a much longer interval between tracer injection and the start of the PET acquisition, thus facilitating enhanced tracer clearance resulting in higher contrast images. Tracers that have been rejected in the past because of slow clearance – and thus too high background signal – could be reassessed, as they may still be suitable tracers if a longer distribution time before the start of the PET acquisition can be applied [24]. In this context, not only radiolabelled small molecules with a slow clearance from circulation are of interest, [25, 26] but also tracers based on small proteins, such as antibody fragments. The clearance rate of antibody fragments increases with the size of the protein [25, 26]. Consequently, the half-life of frequently used isotopes like ^68^Ga and ^18^F is generally too short for PET tracers based on proteins larger than ca. 50 kD. Larger proteins are usually labelled with isotopes with a longer half-life, like ^64^Cu or ^89^Zr. However, the availability of these isotopes is limited. Moreover, ^64^Cu and ^89^Zr cause a much higher radiation burden to the patient, not only because of the long half-life, but also because only about 20% of their radioactive decay occurs via positron emission. With an extended FOV PET camera, images can be acquired at later time points, allowing longer clearance times and thus enabling the use of ^68^Ga and ^18^F as a labelling option for larger proteins, for which previously longer-lived isotopes had to be used [5, 27, 28]. Radiolabelling of peptides and proteins can be easily achieved by conjugation of the peptide or protein with a suitable chelator, followed by complexation with a short-lived radiometal (salt) like [^68^Ga]gallium chloride, or aluminium [^18^F]fluoride. These labelling procedures can be performed under mild conditions that do not compromise the integrity of the peptide/protein. Moreover, these short-lived isotopes are broadly available, can be used in many centres worldwide and have already been used successfully to label peptides and small proteins. Also, when accurate quantitative analysis of PET data by pharmacokinetic modelling of the binding kinetics of a tracer is required, the extended FOV PET camera can be of additional value [29]. This may be particularly relevant for ^11^C-labeled tracers intended for PET imaging of receptors with low expression levels. Because of the low receptor density, tracers with a very high affinity (and high molar activity) are essential to obtain an acceptable target-to-background ratio. Tracers with a high affinity for a particular receptor tend to dissociate slowly from the receptor. In compartmental modelling this is represented by a very small dissociation rate constant (*k*_*4*_ in the two-tissue compartment model). In kinetic modelling studies with ^11^C-labeled tracers, dynamic scans with acquisition time of up to 90 min are usually acquired. Beyond 90 min, the signal-to-noise ratio is usually too poor for proper counting statistics. Because of this time limitation, it can be challenging to properly estimate the dissociation rate constant – and consequently the non-displaceable binding potential (*BP*_*ND*_) – of a tracer with a high receptor affinity. As a result, the kinetics of such a tracer can sometimes be better fitted with an irreversible compartment model than a reversible model. In general, the first part of a dynamic PET scan is dominated by tracer delivery to the tissue of interest, whereas the last part of the scan is more important for the assessment of the receptor binding and dissociation. With extended FOV PET, the acquisition time can be meaningfully prolonged, yielding more data that can be used to assess the dissociation constant. As a result, more reliable estimation of the rate constants – and consequently a more robust estimation of macroparameters like the BP_ND_ – may be obtained for tracers that have previously been discarded, because of slow receptor dissociation.

### Additional requirements for PET tracers

The introduction of the extended FOV camera may offer new opportunities for PET tracers that have been discarded because of slow kinetics. However, extending the tracer distribution time and/or PET acquisition time implies that additional requirements for the properties of the tracer. Most importantly, if the scan will be made at a later timepoint, or data acquisition will be extended, the tracer needs to be sufficiently stable in circulation for a longer period of time. If tracer metabolism is fast, relative to the duration of the tracer distribution/scan acquisition period, no tracer will be available anymore for specific tracer uptake during the extended distribution time. In case the tracer is not very stable for an extended distribution period, its stability can sometimes be improved by replacing one of more hydrogen atoms with deuterium atoms, as has already been shown for several PET tracers [30]. However, the “kinetic isotope effect” by deuteration of the compound is not applicable for stabilisation of all PET tracers, as for example for deuterated [^11^C]deprenyl [31].

### Kinetic modelling may become more challenging

When less radioactivity is injected in a patient, the uptake in blood and metabolite samples is also lowered, whereas the gamma counter to measure the samples retains its original sensitivity.

For this reason, analysis of blood and metabolite samples especially of later time points will become inaccurate as the count rate will get much closer to the background levels. So, for studies using kinetic modelling it is not recommended to inject lower amounts of radioactivity unless more sensitive or rapid analytical methods and/or equipment becomes available.

Another complication of kinetic modelling studies using the extended FOV PET cameras is the fact the patients are less accessible for blood sampling. Consequently, substantially longer lines for arterial blood sampling need to be used, which give rise to more dispersion and adhesion of the PET tracer to the arterial line. The tendency of the tracer to stick to the arterial line generally increases with its lipophilicity. Adhesion of the tracer also depends on the material the line is made of, with polyethylene lines usually showing more adhesion than Teflon lines. Adhesion of the tracer to the injection line can easily be compensated for by measuring the amount of radioactivity that was retained in the line. Compensation for the adhesion of the tracer to the arterial sampling line is more complicated. First of all, blood will be continuously sampled during the scan and retention of the tracer will therefore change over time. Second, the retention of the tracer to the sampling line may be different from the retention of the metabolites to the sampling line. Metabolites are generally less lipophilic than the intact tracer and therefore likely show less retention to the arterial line. As a result, the percentage of intact parent tracer may be underestimated. So, for kinetic modelling studies using an extended FOV PET camera it is even more important to verify that the retention of the tracer to the lines is within acceptable limits.

The high sensitivity and extended FOV PET are, however, promising in allowing the development of more accurate organ-specific kinetic models which may include the metabolite generation in various parts of the body and their incorporation in the circulation. One additional advantage of having an extended FOV scanner is that the input function can be derived more accurately from the image using the left ventricle or the aortas, thus enabling non-invasive kinetic analysis [14]. This consequently can lead to more reproducible quantification of the underlying biology. Such aspects can prove important in the use of radiotracers with interesting kinetic properties in contradiction to what is currently used in the clinical practice, even for [^18^F]FDG [32]. Furthermore, the calculation of the arterial input function from various parts of the body may make redundant the need for arterial catheterization and allow organ-specific input function accommodation for the effects of delay and dispersion directly from the measured data [33].

### High costs of an extended FOV camera

The extended FOV PET cameras that are currently available are much more expensive than regular PET-CT cameras. These costs might be compensated for if the camera can be used as a high throughput system for regular patient care. Usually, business cases for the acquisition of an extended FOV PET camera are based on the fact that more patients can be scanned, because of the shorter acquisition times [11]. Usually, the situation that scans may take longer as a result of a lower injected dose or longer waiting time after injection is not taken into account. Researchers that aim to depopulate the graveyard of failed PET tracers that were discarded because of too slow tracer kinetics should realize this. Revival of these discarded tracers may be an interesting goal from a scientific point of view, but the economic side-effect is enormous. Extending the acquisition time of an extended FOV PET camera to the acquisition times that are regularly used with a conventional PET camera could easily increase the price of a PET scan with a factor 3–5. The question is whether the revived tracers are good enough to justify these extra costs and whether patients and healthcare insurances are willing to pay for this. The development of inexpensive extended FOV PET with new lower cost elements may have a critical role in the coming years [34].

## Concluding remarks

With the emerging opportunities and especially the increased sensitivity accompanying the extended FOV PET-cameras, there are several new possibilities for PET-tracers development both in the radiochemistry development as in the imaging and clinical application of these radiopharmaceuticals. Besides the potential revival of failed radiopharmaceuticals, novel challenges are ahead to develop novel radiochemistry based on thus far unsuitable (low yielding or time consuming) reactions.

To fully benefit from the reported advantages also innovation is required for the supporting technology, equipment and infrastructure.

## Data Availability

Datasets mentioned in this article can be found in the cited articles.

## Abbreviations

A_m_: Molar activity
CT: Computed Tomography
FAP: Fibloblast Activating Protein
FDG: [^18^F]Fluoro-2-deoxyglucose
FOV: Field of View
kD: Kilo Dalton
LAFOV: Long axis Field of View
PET: Positron Emission Tomography
PSMA: Prostate Specific Membrane Antigen
SPECT: Single Photon Emission Computed Tomography
